# Mesenchymal stem cells and dental implant osseointegration during aging: from mechanisms to therapy

**DOI:** 10.1186/s13287-023-03611-1

**Published:** 2023-12-20

**Authors:** Yang Ma, Siyuan Wang, Hui Wang, Xiaoyu Chen, Yi Shuai, Huiming Wang, Yingjie Mao, Fuming He

**Affiliations:** 1 Stomatology Hospital, School of Stomatology, Zhejiang University School of Medicine, Zhejiang Provincial Clinical Research Center for Oral Diseases, Key Laboratory of Oral Biomedical Research of Zhejiang Province, Cancer Center of Zhejiang University, Hangzhou, China; 2 Nanjing Jinling Hospital: East Region Military Command General Hospital, Nanjing, China

**Keywords:** Aging, Osseointegration, Mesenchymal stem cells, Differentiation, Molecular mechanism

## Abstract

Dental implants are widely used to replace missing teeth, providing patients with unparalleled levels of effectiveness, convenience, and affordability. The biological basis for the clinical success of dental implants is osseointegration. Bone aging is a high-risk factor for the reduced osseointegration and survival rates of dental implants. In aged individuals, mesenchymal stem cells (MSCs) in the bone marrow show imbalanced differentiation with a reduction in osteogenesis and an increase in adipogenesis. This leads to impaired osseointegration and implant failure. This review focuses on the molecular mechanisms underlying the dysfunctional differentiation of aged MSCs, which primarily include autophagy, transcription factors, extracellular vesicle secretion, signaling pathways, epigenetic modifications, microRNAs, and oxidative stress. Furthermore, this review addresses the pathological changes in MSCs that affect osseointegration and discusses potential therapeutic interventions to enhance osseointegration by manipulating the mechanisms underlying MSC aging.

## Introduction

The rapid increase in the aging population has become a concern worldwide; the global population aged ≥ 65 years is estimated to reach approximately 1.5 billion by 2050 [1]. With a surge in the aged population, an increased number of patients will face dental issues, requiring tooth replacement [2]. Tooth loss not only impairs the ability to chew and grind food but also makes it difficult to speak and modifies the facial features [3]. For decades, dental implants and implant-supported prostheses have been clinically applied as the most effective methods for oral rehabilitation of partially or fully edentulous patients. Notably, the long-term survival rate is 93.3–97% [4, 5]. The prerequisite for clinical success of dental implants is osseointegration, defined as a direct structural and functional connection between the bone and the surface of the implant [6, 7]. Elderly patients undergoing implant surgery are at higher risk compared to younger patients owing to their age-related health conditions [8]. With an increase in age, bone quality and quantity deteriorate, leading to osteoporosis [9]. Senescent bone marrow mesenchymal stem cells (BMMSCs) have impaired osteogenic differentiation and increased adipogenic differentiation abilities and play key roles in bone aging [10]. Differences in bone metabolism and biological properties of MSCs between elderly and young patients could result in differences in osseointegration patterns and success rate. The present review aims to provide an overview of the existing literature on the molecular mechanisms of MSC and bone aging and their effect on osseointegration. Based on this information, possible techniques to enhance osseointegration in aged population are discussed.

### Osseointegration and aging

In the 1960s, Professor P. I. Branemark laid the foundation of modern implant dentistry by discovering the phenomenon of direct bone-to-implant contact; he termed it as “osseointegration” [6]. During the same period, Professor Andre Schroeder reported direct bone-to-implant contact and soft tissue reaction to titanium [11, 12]. Osseointegration is the direct structural and functional connection between the dental implant surface and the living bone without intervening soft tissue.

**Fig. 1:**
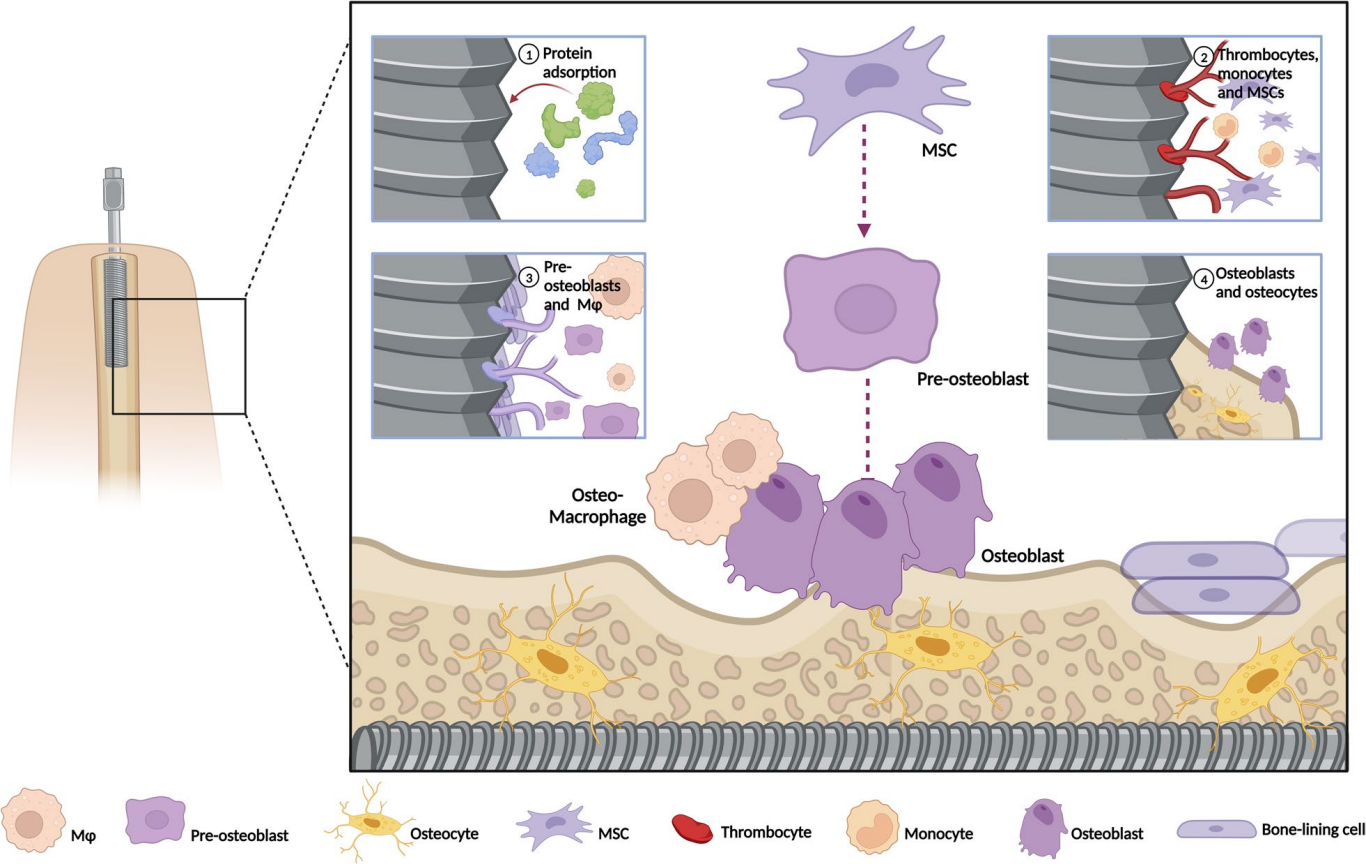
Schematic representation of the osseointegration process. Implant placement triggers the following events to initiate osseointegration. Titanium implant surface causes protein adsorption, followed by coagulation and complement system activation. Monocytes differentiate into macrophages and control the immune response; MSCs are recruited in an accurate balance and commit to bone-forming cells, leading to bone formation. Created with http://BioRender.com

Titanium implants need to go through a cascade of healing events before they are “accepted” by the body tissues. The implantation of biomaterials results in injury and initiation of the inflammatory response regardless of the method of introduction of the biomaterial into the body [13, 14]. An immune response occurs, as biomaterial insertion leads to the disruption of the host’s tissue [15]. Accordingly, osseointegration can be perceived as an immune-modulated inflammatory process, wherein the immune system is locally regulated, thus influencing the whole healing process. The osseointegration process includes several phases post implant placement. Initially, titanium implant surface causes surface protein absorption, followed by coagulation and complement system activation. Then, monocytes are recruited and differentiate to macrophages to modulate the immune response. MSCs are recruited and differentiate to osteoblasts and osteocytes to deposit collagen matrix and form new bone. Finally, the peri-implant interface is completely replaced by a mature lamellar bone [16, 17] (Fig. 1).

Bone aging is characterized by decreased osteogenesis and increased adipogenesis[10]. Whether bone aging is a negative factor in implant osseointegration has gained interest among the research communities. However, studies have reported contrasting results regarding the success and failure of dental implants in both patients and animals with bone aging. It has been reported that patients with advanced age receiving dental implants could have excellent implants survival rates and low periodontal disease index scores. A systematic review and meta-analysis reported high implant survival rates in patients aged over 75 years (97.3% for 1 year and 96.1% for 5 years) [18]. In 1998, 39 older patients who had 190 implants supporting 45 oral prostheses and 43 younger adults who had 184 implants supporting 45 oral prostheses were compared after being monitored for a period of 4–16 years. The data showed an implant success rate of 92.0% for the older group compared to an success rate of 86.5% for the younger group; however, no statistical significance was observed [19]. Moreover, a prospective study concluded that the clinical performance of mandibular two-implant overdentures is equally successful in younger and older patients after 10 years [20]. In contrast, Alsaadi et al. reported a positive relation between osteoporosis and early implant failure in a retrospective study [21]. Similarly, a systemic review concluded that osteoporotic subjects presented higher rates of implant loss [22]. Moreover, Niedermaier et al. studied survival rates of implant-supported dentures in patients with osteoporosis for up to 7 years and revealed increased rates of implant failure in these patients [23]. A multicenter study reported the lowest success rate in older patients after 6 years for 1022 implants placed in 440 patients [24]. Bertl et al. [25] reported that patients ≥ 80 years old showed a higher rate of early implant loss than younger patients. A retrospective, cross-sectional, matched sample study concluded that osteoporosis has a significant influence on peri-implant marginal bone level at the mesial and the distal implant aspect in postmenopausal women [26]. An animal study revealed that age-related estrogen deficiency in rats negatively influences preexisting bone around titanium implants in both the cortical and cancellous bones [27]. Collectively, it can be concluded that the osseointegration process is compromised in aged individuals, which may cause implant failure. Therefore, therapies enhancing the osseointegration process in the aged population should be developed.

### Strategies to improve osseointegration in aged individuals

Various methods have been used to improve osseointegration in the aged population. These include systemic and local application of certain drugs and modification of the implant surface through novel techniques, such as varying the topography, applying coatings, or combining both of these. Systemic administration of bone-regulating hormones, such as calcitonin, parathyroid hormone (PTH), and estrogen, significantly improved bone formation and implant anchorage in osteoporotic rats [28–31]. Strontium ranelate and simvastatin improve titanium implant osseointegration by promoting bone formation and inhibiting bone absorption through various signaling pathways [32]. Currently, the common sand blasting and acid etching strategy is widely applied to increase the surface roughness of implants, which enhances biological properties of MSCs and osseointegration process [33]. In addition to the design of implant surface, it is necessary to promote osteoinduction and inhibit bone resorption locally in osteoporotic patients. Implants coated with bioactive agents, such as anti-osteoporosis drugs [34, 35], bioactive molecules [36, 37], or bioactive inorganic elements [38, 39] with proper controlled release have been reported to be able to improve osseointegration in osteoporotic individuals. Although numerous methods have been developed to achieve favorable osseointegration in osteoporotic conditions, the exact underlying biological mechanisms of how bone aging affects the osseointegration process and possible methods to improve osseointegration based on molecular mechanisms of bone aging have not been summarized before.

### MSCs in bone aging

**Fig. 2:**
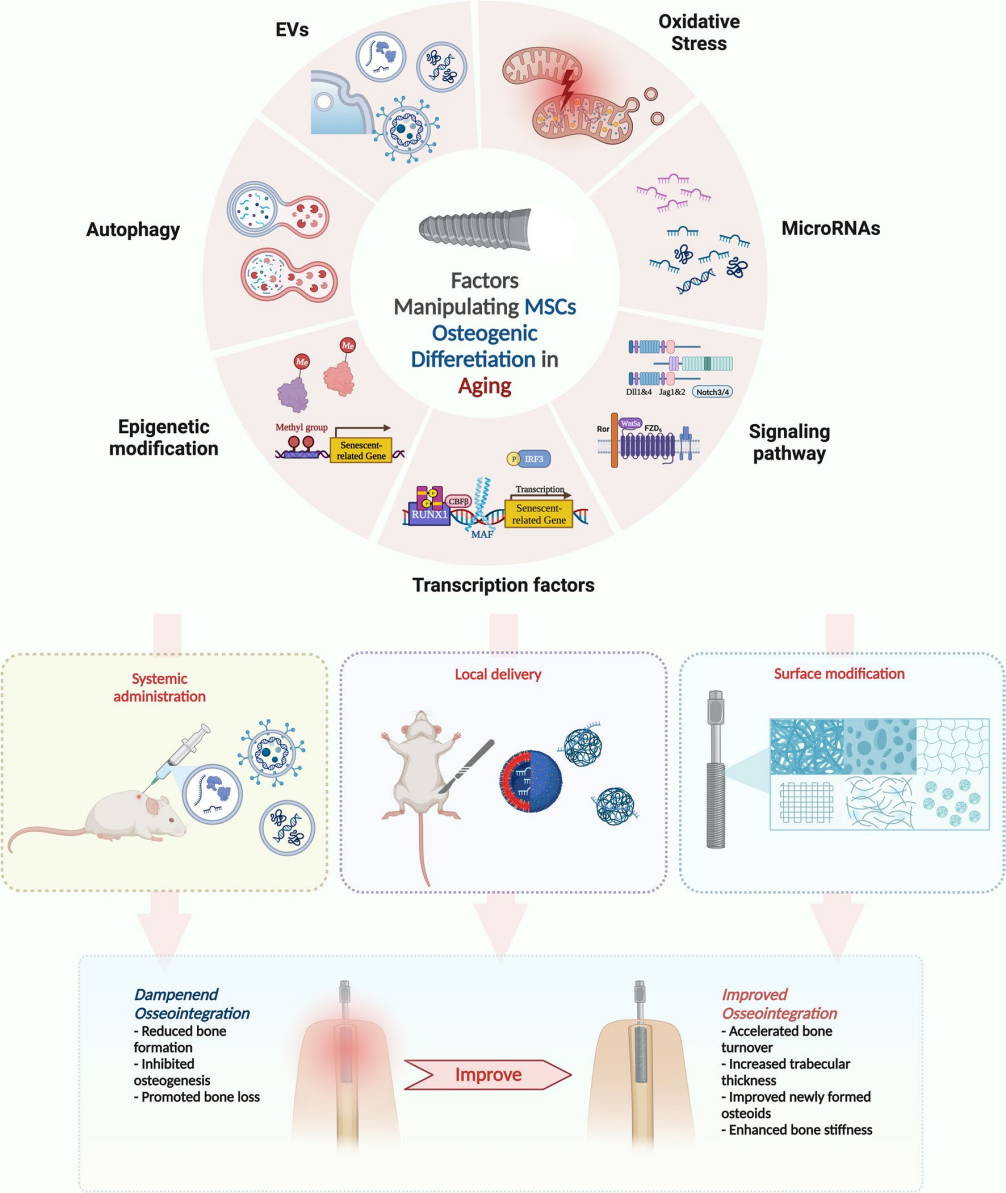
Schematic representation of the potential uses of the mechanisms of MSC aging in improvement of osseointegration. Molecular changes associated with age-related changes in MSCs from varying perspectives include autophagy, transcription factors, EV secretion, signaling pathways, epigenetic modifications, microRNAs, and oxidative stress. Possible interventions at the molecular level to improve osseointegration include systemic administration, local delivery, and surface modification. Created with http://BioRender.com

MSCs are mesoderm-derived progenitor cells that can be obtained from a wide range of tissues such as bone marrow, adipose tissue, umbilical cord, muscle, and dental tissue. They have the ability to self-renew and differentiate into various mesodermal cell types, including osteoblasts, adipocytes, and chondrocytes [40]. During osseointegration, followed by MSCs recruitment to the bone remodeling site, these cells proliferate and commit to pre-osteoblasts, further maturing into osteoblasts, which are involved in initial matrix secretion, maturation, and mineralization. At the end of the bone-forming stage, osteoblasts can have one of the following fates: develop into osteocytes embedded in the mineralized bone matrix, inactivate to quiescent bone-lining cells, or undergo apoptosis (Fig. 1) [16, 17, 41]. During bone aging, BMMSCs population declines and show reduced osteogenic differentiation and increased adipogenic differentiation [42, 43]. This could be a possible reason for impaired osseointegration and implant failure. However, the signaling pathways driving this pathological shift remain elusive and are currently under thorough investigation. Understanding the molecular mechanisms governing the dysregulated osteogenesis of aged MSCs is crucial for developing new treatments to promote bone formation and enhance osseointegration. Here, we summarize the pathological and molecular changes governing the biased differentiation of BMMSCs from various aspects, including autophagy, transcription factors, EV secretion, signaling pathways, epigenetic modifications, microRNAs, and oxidative stress. Furthermore, the effects of pathological changes in MSCs on osseointegration and possible interventions at the molecular level, such as modifications of implants and their utility in treatment-based applications, are discussed (Fig. 2).

### Osseointegration and aging: lessons from MSCs

#### Autophagy

Autophagy is a cellular process through which redundant components, such as proteins and damaged mitochondria, are engulfed and delivered to lysosomes for degradation and recycling to maintain cellular homeostasis [44]. Reduced autophagy is a hallmark of aging in both cells and tissues. The expression of several autophagy-related genes is significantly reduced in various aged cells and tissues compared to that in their younger counterparts [45, 46]. Activation of autophagy by rapamycin, an inhibitor of the mTOR pathway, has been shown to increase the life expectancy of mice when fed them to late in life [47] or for a short interval during mid-life [48]. BMMSCs from aged mice present impaired autophagy compared to those from young mice. Furthermore, stimulation of autophagy by rapamycin not only enhances the osteogenic differentiation of aged BMMSCs in vitro but also restores the bone mineral density of senile osteoporotic aged mice [49].

Recently, it was revealed that during the process of implant osseointegration, several modifications in the topography of dental implants could promote osseointegration by modulating autophagy of the surrounding cells [50, 51]. Furthermore, metformin treatment promotes implant osseointegration under osteoporotic conditions by increasing autophagy and the osteogenesis of MSCs [52]. Moreover, multifunctional hydrogels fabricated from dynamic crosslinking of synthetic polymers, natural polymers, and silver nanowires to deliver rapamycin significantly improve osseointegration in vivo and restore degenerative cellular properties of BMMSCs in vitro in osteoporotic models by increasing autophagy [53]. Collectively, the up-regulation of autophagy by different methods, including implant modification and drug application, could be a promising mechanism to enhance osseointegration in the aged population.

#### Transcription factors

Numerous transcription factors have been identified as critical mediators involved in the aging process and osteogenic differentiation [54]. Recent studies have shown that the expression of transcription factors, including FOXP1, MAF, CBFβ, and SATB2, could potentially affect and attenuate senescence-related changes, including reduced bone mineral density, decreased trabecular thickness, and increased bone marrow adipogenic differentiation [55–58]. In this context, elucidation of the links between transcription factors and osseointegration may aid in understanding bone metabolism, optimizing aging-relevant skeletal disease therapies, and broadening the applications of dental implants.

##### FOXP1

Forkhead box P1 (FOXP1) is a critical transcription factor that participates in and affects multiple biological processes. It plays a vital role in defining the switch of MSCs from osteogenic to adipogenic differentiation by binding to the proliferator-activated receptor γ2 (PPARγ2) promoter [59, 60]. Li et al. recently reported age-correlated FOXP1 declination in BMMSCs as a cause of up-regulated adiposity and down-regulated bone mass, which may function through the inhibition of p16 [61]. Specifically, conditional ablation of FOXP1 in MSCs is associated with up-regulation of the CEBPβ/δ complex and dysregulation of recombination signal binding protein for immunoglobulin κ J region (RBPjκ), which further results in a reduced regenerative capacity of MSCs in vivo [55]. These studies preliminarily demonstrate the role of FOXP1 in regulating the fate of MSCs and age-dependent bone metabolism [62]. However, further studies are required to elucidate the underlying mechanism connecting FOXP1 and osseointegration.

##### MAF

MAF bZIP transcription factor (MAF) is a regulatory factor in the development of multiple tissues and immune system. Additionally, it is involved in senescence-dependent MSC differentiation [10]. Nishikawa revealed the age-dependent declination in Maf expression in murine MSCs, which affected the osteoblast/adipocyte bifurcation in MSCs by interfering with the osteogenesis mediator Runx2 and the adipogenesis regulator Pparg. [58]. Furthermore, strontium can attenuate age-related bone loss, characteristic of decreased bone mineral density and trabecular thickness, by targeting Maf [63, 64]. Similarly, Zn2 + /Sr2 + -collagen co-assembly with hydroxyapatite (HA) promotes bone reconstruction by up-regulating Nfatc1/Maf signaling pathway [65]. These results suggest Maf as a promising target to allocate the lineage of MSCs by inhibiting aging-dependent switching of osteoblasts to adipocytes and could be applied in the process of implant osseointegration in individuals with osteoporosis.

##### CBFβ

Core binding factor subunit beta (CBFβ) serves as a pivotal transcription factor in regulating osteoblast differentiation, bone anabolism, and senescence-related skeletal development by stabilizing and promoting Runx family proteins [56, 66–68]. Along with substantial accumulation of bone marrow adipocytes and reduced bone mineral density, Cbfβ expression is dramatically diminished in aged mice, indicating that Cbfβ plays a critical role in age-related bone anabolic metabolism [69]. Furthermore, Wu et al. reported that Cbfβ manipulates osteogenesis/adipogenesis lineage commitment by activating the Wnt10b/β-catenin signaling pathway and inhibiting adipogenesis regulatory gene (c/ebpα) expression [69]. In addition, conditional abrogation of Cbfβ in BMMSCs significantly impairs osteogenic capacity and reduces bone mineral density [69, 70], whereas its overexpression can certainly reverse senescence and adipogenesis mediated by p53/miR-145a [71, 72]. In conclusion, these studies primarily unveil the regulatory role of CBFβ in osteoblast/adipocyte lineage commitment, which could be exploited as a therapeutic target for promoting osseointegration in aged individuals.

##### SATB2

Special AT-rich sequence-binding protein 2 (SATB2), a nuclear matrix protein, is a pivotal mediator in coordinating stemness maintenance, craniofacial skeletal patterning, and osteogenic differentiation by H19 [73], miR-31 [74, 75], miR-103 [76, 77], miR-140-5p [78], and MALAT1 [79]. Interestingly, SATB2 expression decreases during senescence in alveolar bone-derived BMMSCs [80], through the regulation of Nanog transcription [81, 82]. In parallel with SATB2 declination, the BMMSCs derived from elderly rats exhibit abrogated osteogenesis and up-regulated adipogenesis [83]. However, exogenous overexpression of SATB2 decreases the age-related changes in MSCs, thereby reversing senescence-related alveolar bone loss [81] and facilitating bone reconstruction in critical size mandibular defects [84]. In addition, local delivery of SATB2 significantly accelerates new bone formation and promotes the osseointegration of titanium implants in vivo [57]. Considering this, increased SATB2 expression could be an effective strategy to regulate bone osteointegration and manage age-related bone diseases.

#### Extracellular vesicles (EVs)

##### Exosomes

**Fig. 3:**
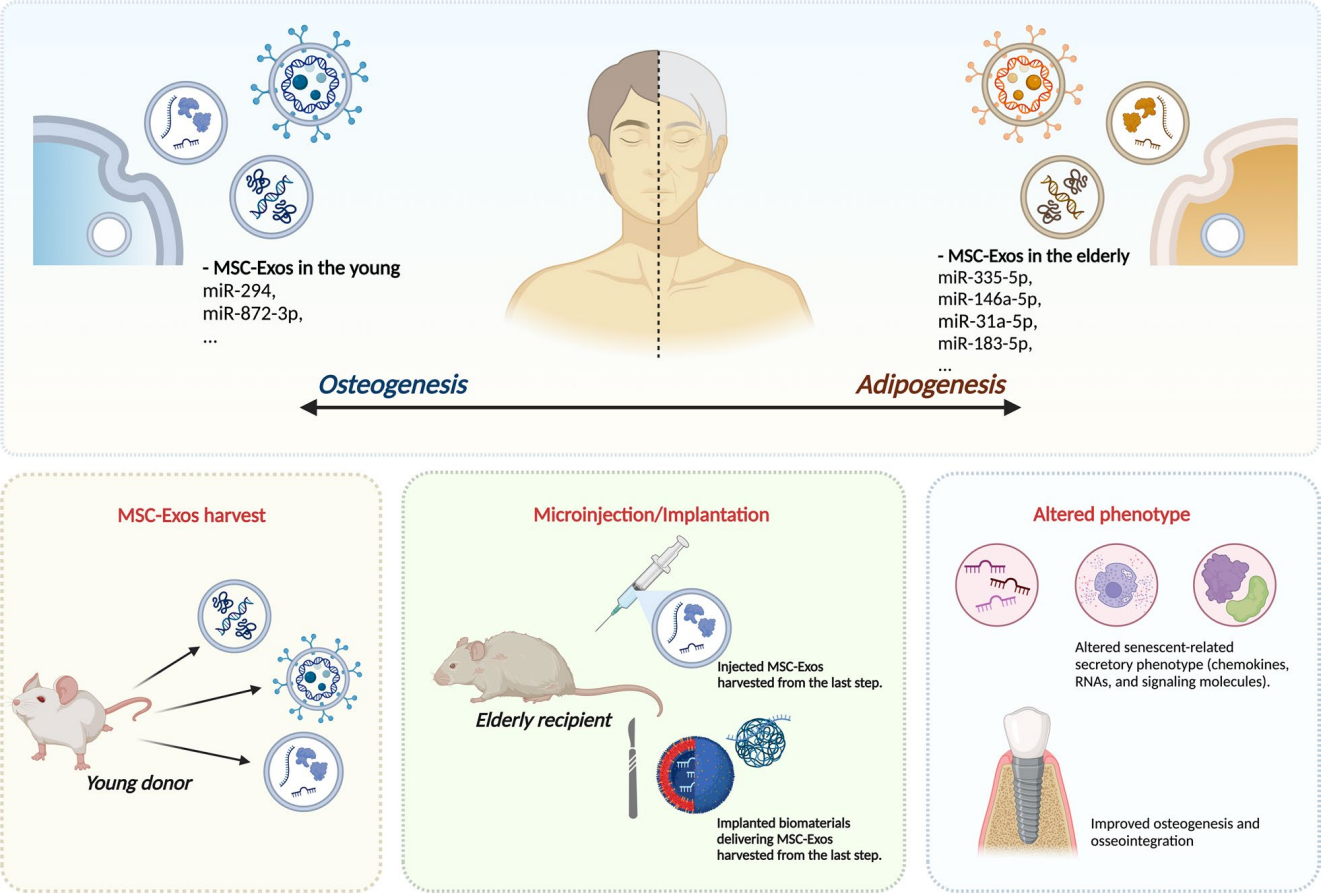
Schematic representation of exosome application from young individuals for improving osseointegration with aging. The expression pattern of bone marrow exosomes differs significantly between the young and elderly. The application process includes MSC-Exo harvest, local application, and implantation. Created with http://BioRender.com

EVs are membrane nano- and micro-vesicles generated by various cells and organs (MSCs [85], DCs [86], B cells [87], mast cells [88], epithelial cells [89]), which are important in cell-to-cell communication [90] and multiple biological processes [91, 92]. Among them, MSC-derived exosomes containing proteins, RNAs, and lipids serve as key mediators of the aging process [93]. The expression pattern of bone marrow exosomes differs significantly between the young and elderly [94]. For instance, exosomal presentation of miR-294 and miR-872-3p [95] is significantly reduced during senescence, while bone marrow expression of miR-335-5p and miR-146a-5p [96] is characteristic in MSC-derived microvesicles secreted by older rats. The expression of exosomal miR-31a-5p, serving as a critical modulator of osteoclastogenesis and bone resorption, is markedly higher in aged MSCs than in young cells. Inhibition of miR-31a-5p expression prevents bone loss and reduces the osteoclastic activity of aged rats in the bone marrow microenvironment [83]. Intercellular transfer of microvesicles from young MSCs rejuvenates aged murine hematopoietic stem cells (HSCs) [97]. In addition, incubation of young MSCs with aged exosomes (including miRNA-183-5p) inhibits MSC differentiation into osteoblasts, thereby inhibiting osteogenesis [98]. In contrast, exosomes highly expressing miRNA-19b-3p from young donors ameliorate the reduced osteogenic differentiation of BMMSCs in aged rats with osteoporosis in vivo [99]. The exosomes derived from young BMMSCs specifically containing CD9, CD63, and TSG101 promote new bone regeneration and osseointegration during distraction osteogenesis (DO) in older rats [100]. Collectively, these results indicate that the exosomes derived from older MSCs are associated with reduced osseointegration. In terms of sustained release and durable efficacy, liposomes [101], nanohydroxyapatite (nHP) [102, 103], tricalcium phosphate (β-TCP) ceramics [104, 105] and hydrogel formulations (e.g., alginate [106, 107], hyaluronic acid [106, 107], poly (lactic-co-glycolic acid) (PLGA) [108–110], and polydopamine (PDA) [109, 110]) have been used to deliver MSC-Exos for osteogenesis and osseointegration. Recently, micro/nano-textured hierarchical titanium topography has been shown to be favorable for BMMSC-Exos biogenesis and secretion, as it promotes osseointegration [111]. These findings suggest that remodeling MSC-Exos via surface modification of biomaterials or local delivery may provide a cell-free strategy for enhancing osseointegration (Fig. 3). However, the efficacy of this method requires further validation.

##### Apoptotic vesicles

Apoptotic vesicles (ApoVs) are a heterogeneous population of EVs generated during apoptosis [112]. Reduction of apoptotic body formation significantly impairs the self-renewal capacity and the balance of BMMSCs between osteogenesis-or-adipogenesis [113]. Young MSCs-derived apoVs effectively rejuvenate aged BMMSCs by enriching the levels of Ras-related protein 7 (Rab7) [112]. Previous studies have shown apoVs to be effective in bone homeostasis, as they regulate bone remodeling [114, 115]. Nevertheless, further investigation on the design and use of MSC-Exos, especially MSC-apoVs, can potentially present a unique opportunity for developing strategies for osseointegration enhancement and bone regeneration in the future [116].

#### Intracellular signaling pathways

Multiple signaling pathways regulate bone homeostasis and bone cell differentiation. Therefore, targeting signaling pathways to enhance the osseointegration process is feasible. Here, we describe the pathways that are known to be affected by age and result in biased differentiation of MSCs.

##### Wnt signaling

Wnt signaling is essential for differentiation of MSCs and bone metabolism. Recently, it has been shown to be associated with aging. In the canonical Wnt pathway, Wnt proteins bind to their respective dimeric cell surface receptors, comprising seven transmembrane frizzled proteins and the LRP5/6, to activate the signaling cascade by recruiting, phosphorylating, and activating the cytoplasmic protein disheveled (DVL). This in turn inhibits the phosphorylation of β-catenin by the complex (GSK-3, APC, and Axin) and subsequently, promotes the translocation of stabilized β-catenin into the nucleus to stimulate target gene expression [117]. Zhang et al. reported that Wnt/β-catenin signaling is highly activated during MSC aging, leading to DNA damage response and p53/p21 pathway activation by regulating reactive oxygen species (ROS) production [118, 119]. Another study showed that the expression of various Wnt-associated genes decreased in the bone tissues of aged mice compared with that in the tissues of young mice [120]. Kathleen et al. performed RNA-seq of implant-associated tissue from old and young mice one week after implantation and revealed that the expression of several Wnt ligand receptors (Fzd4, 5, 8, and 9) and Wnt-regulated transcription factors (Tcf7l1, Tcf7l2, Tle2, and runx2) decreased in old mice [121]. Wnt-10b is essential to maintain normal bone density and mesenchymal progenitor activity in adult bone. Wnt-10b-/- mice show age-dependent loss of bone mass and a reduced number of MSCs. However, transgenic overexpression of Wnt-10b prevents bone loss in aged mice and enhances osteogenesis in vitro [122]. In addition, ROS activate FoxOs, which in turn, binds β-catenin to reduce its availability for activating osteogenesis-related transcription factors, leading to slow bone formation [123, 124]. A recent study revealed that Gli1 + cells contribute to the formation of new bone around the dental implant and that the ablation of these cells impairs osseointegration. Canonical Wnt signaling plays crucial roles in mediating Gli1 + stem cells [125]. Collectively, Wnt signaling mediates bone aging process by acting at different levels. Thus, modulating Wnt signaling could be a promising direction for improving osseointegration.

##### Notch pathway

Notch pathway is an essential regulator of bone development. Notch signaling is crucial for the maintenance of MSCs pool under physiological conditions. Disruption of Notch signaling causes bone loss in aged mice and increased trabecular bone mass in adolescent mice [126]. RNA-Seq of peri-implant tissue from young and old mice reveals multiple genes, including Notch ligands (Jag1, Jag2, Dll1, and Dll4), receptors (Notch 3, Notch 4), and downstream transcription factors (Hey1, Hey2, Heyl, and Hes1), in the Notch pathway; the expression of these genes are significantly decreased in old peri-implant tissue. This indicates that Notch signaling is inhibited during aging after implantation [121] and that targeting the Notch pathway may enhance osseointegration in older patients. However, another study showed that the Notch pathway is activated in aged BMMSCs, and inhibition of the Notch signaling by γ-secretase inhibitor restores the osteogenic ability of BMMSCs [127]. Nevertheless, 12-month-old C57BL/6 mice were used as the old group in this study, and only an in vitro study was conducted, whereas the previous study was performed on 21-month-old mice as the old group in vivo. These differences may be responsible for the varying results. Notch activation by Jagged 1 enhances the osteogenic differentiation of MSCs sheet by inhibiting the cellular senescence caused by high density sheet culture [128]. Therefore, Notch pathway could be a key regulator of MSCs and bone aging. However, the role of Notch signaling in osseointegration has not been thoroughly explored. Local aspirin administration enhanced hydroxyapatite-coated titanium implant osseointegration in OVX rats through activation of the Notch pathway in osteoporotic bone [129]. Notch pathway is activated by chemical/nanostructural (modSLA) and micro-roughened (SLA) surfaces that lead to increased osteogenic differentiation after culturing in osteogenic media [130]. In summary, Notch signaling is closely associated with bone and MSC aging, and its modulation might be an effective strategy to enhance osseointegration in the elderly.

##### NF-κB signaling

The transcription factor nuclear factor κB (NF-κB) is a key regulator of inflammation and bone-modeling process. Increased NF-kB activity is accompanied by increased bone-resorption and decreased bone-formation abilities [131]. The MSCs derived from aged mice show impaired osteogenesis, which is associated with increased NF-κB activity [132]. The proinflammatory cytokines TNF and IL-17 inhibit the osteogenic differentiation of MSCs by activating NF-κB signaling. Inhibition of NF-κB signaling promotes bone regeneration and repair under chronic inflammatory conditions [133]. Prophylactic melatonin administration promotes the osteogenesis of BMMSCs and osteoblasts in vitro and reduces bone resorption and proinflammatory cytokine levels by inhibiting the activation of NF-κB to down-regulate TNF, IL-1β, and IL-6 [134]. These findings provide an unexplored strategy for enhancing osseointegration by inhibiting the NF-κB pathway. Methods such as drug administration to inhibit NF-κB signaling should be further investigated.

#### Epigenetic modification

The role of epigenetic alterations in aging has become an interesting topic owing to the reversibility of these alterations. This may introduce a scope for developing treatment options for age-related diseases [135]. Epigenetic modifications such as DNA methylation, histone modification, and chromatin remodeling regulate the patterns of gene expression by altering DNA accessibility or chromatin structure without changing the DNA sequence. They are both heritable and reversible and can occur in response to environmental stimuli and intrinsic changes to maintain cell homeostasis and function [136]. It has been widely documented that epigenetic changes play crucial roles in the aging process of MSCs. Several regulatory factors and sites related to DNA methylation have been found to have profound effects on MSC aging. Kalyan et al. compared the methylomes of young and aged human BMMSCs using sequencing-based methods, identified DNA methylation changes associated with aging, and constructed hypo- and hypermethylation-specific regulatory networks [137]. RG108, the inhibitor of DNA methyltransferase (DNMT), can alleviate the senescence of aged human BMMSCs by eliminating ROS and upregulating telomerase traverse transcriptase (TERT) activity, which can repair shortened telomeres [138]. Depletion of the DNA demethylases TET1 and TET2 leads to an osteopenia phenotype in mice. Moreover, it reduces the self-renewal and osteogenic differentiation abilities of BMMSCs by inhibiting demethylation of the P2rX7 promoter and the release of exosomes, leading to the accumulation of miR-297a-5p, miR-297b-5p, and miR-297c-5p in BMMSCs, which inhibits the Runx2 signaling pathway [139]. Histone modifications regulate aging in MSCs by regulating the transcriptional activity of the related genes. For instance, Twist-1 is down-regulated in aged MSCs, whereas overexpression of Twist-1 alleviates human MSC senescence by increasing the recruitment of EZh2, reducing the expression of the Lnk4A/Arf locus, and enhancing the levels of histone H3K27me3 at p16/p14 promoters [140]. In osteoporosis models, epigenetic modifying protein lysine (K)-specific demethylase 5A (KDM5A) is up-regulated, which in turn suppresses runx2 expression by reducing H3K4me3 levels [141]. Similarly, the expression of enhancer of zeste homology 2 (EZH2) increases in osteporotic MSCs. It directly elevates H3K27me3 levels on the promoters of Wnt1, Wnt6, and Wnt10a to silence Wnt signaling. Knockdown of EZH2 and inhibition of H3K27me3 inhibit the repression of Wnt signaling and restore the osteogenic differentiation of osteoporotic MSCs [142]. However, absent, small, or homeotic-like 1 (Ash1l), a histone 3 lysine 4 (H3K4) trimethyltransferase in mice, is downregulated and fails to mediate H3K4me3 recruitment at the transcription start sites of the OSX, Runx2, Sox9, and Cred genes. Silencing of Ash1l impairs osteogenic differentiation and promotes adipogenic differentiation [143]. In addition, microRNAs participate in the epigenetic regulation of MSC aging and osseointegration. This is further elaborated in the following section.

The involvement of epigenetic modifications in osseointegration and their application in functionalizing implant surface or systematically improve osseointegration need to be further investigated. To date, only the effects of microRNA coatings on implant surface have been investigated; DNA methylation and histone modifications have not yet been studied, although they could hold some potential in enhancing the osseointegration process.

#### MicroRNAs

MicroRNAs (miRNAs) are single-stranded noncoding RNAs (∼22 nucleotides long) involved in the repression of the expression of target genes via either mRNA degradation or translational inhibition in a broad range of organisms [144]. miRNAs participate in controlling cell function by acting on more than one gene [145]. Several miRNAs are closely related to age-related changes in MSCs and bone aging. miRNA-195 targets the 3′ untranslated region of TERT. During skeletal aging, MiR-196a was found to be up-regulated in MSCs that targeted homeobox B7 (HOXB7). A forced HOXB7 expression in MSCs led to increased cell growth, reduced senescence, and improved osteogenic differentiation [146]. MiR-188 is a crucial regulator of the age-related differentiation imbalance in MSCs. Its expression is elevated in MSCs derived from aged mice and humans, and forced expression of miR-188 causes age-related bone loss and fat accumulation. It directly targets histone deacetylase 9 (HDAC9) and RPTOR- independent companion of MTOR complex 2 (Rictor) [147]. MiR-141 is up-regulated in the bones and MSCs of mice and humans. It inhibits the osteogenesis of MSCs. It targets ZMPSTE24, BMI1, SDF-1, SVCT2, and DLX5, which are known to control MSCs differentiation, migration, and proliferation [148–151]. Additionally, the expression of microRNAs is reduced in aged MSCs. The expression of MIR17–92 is reduced in old mice, and miR-17 overexpression restores the osteogenesis of old MSCs by regulating Smurf1 [152]. Both microRNA 23a and microRNA 23b are remarkably down-regulated in the MSCs of old mice and humans and are closely related to the imbalanced differentiation of aged MSCs. Overexpression of miR-23a/b promotes the osteogenic differentiation of MSCs, which targets transmembrane protein 64 (TMEM64) [153].

Multiple microRNAs regulate the osseointegration process by controlling the osteogenesis of MSCs [154]. MiRNA and anti-miRNA delivery has been used to improve the osseointegration process by applying them to the implant surface or surrounding tissue of the implant. An miR-21 nanocapsule immobilized on titanium surface via an in situ polymerization method enhanced both the osteogenesis and angiogenesis of MSCs in vitro and improved osseointegration during early stages in vivo [155]. Similarly, Zhen et al. fabricated a titanium (Ti)-based SrHA/miR-21 composite coating via hydrothermal deposition of SrHA, followed by miR-21 nanocapsule immobilization, and revealed that the coating not only increased osteoblast proliferation and differentiation in vitro but also enhanced osseointegration and bone-implant bonding strength in vivo [156]. A microporous Ti implant surface formed by microarc oxidation (MAO) loaded with miR-29b and antimiR-138 via lyophilization enhances the osteogenic differentiation of MSCs and potentially leads to rapid and increased osseointegration of the clinical implant interface [157]. These observations indicate that the application of age-related microRNAs to titanium surface could be a promising direction to enhance osseointegration in the aged population. Further research is necessary for optimizing the process.

#### Oxidative stress

ROS are chemically reactive chemical species containing oxygen, which are mostly generated within the electron transport chain of mitochondria. An increase in ROS levels is highly correlated with aging and damage to cells and tissues [154, 158]. Aging can shift the differentiation preference of MSCs from osteogenic to adipogenic through oxidative stress [159–161]. Consistent studies have shown that treatments eliminating ROS through various mechanisms can alleviate bone aging, and promote bone formation. SIRT3, a sirtuin involved in aging and the overexpression of SIRT3 in MSCs, restores the capacity of MSCs to differentiate and reduces oxidative stress [162]. Similarly, rapamycin enhances the osteogenesis of aged MSCs by restoring autophagy and reducing levels of ROS [49]. Recent research has revealed that titanium surfaces coated with ROS-responsive gelatin/chitosan hold the potential to promote the osteogenic differentiation of MSCs and enhance osseointegration by eliminating intracellular ROS in osteoporotic conditions [163]. Therefore, reducing oxidative stress via application of drugs or implant modifications could be a promising strategy for implant treatment in aged population.

## Conclusions and prospects

The pathological differentiation of senescent MSCs contributes to bone aging, which is detrimental for the osseointegration process and implant success. In this review, we summarized the current knowledge of molecular mechanisms underlying impaired MSCs differentiation in aging and the methods to counteract this imbalanced differentiation to improve osseointegration. In the field of dental implantology, it's evident that the journey toward achieving better osseointegration is multifaceted. This review provides new perspectives and potential strategies to enhance osseointegration and increase implant survival rate. However, osseointegration process include a cascade of events involving different kinds of cells and we still lack the knowledge of the cellular and molecular mechanisms of osseointegration during aging. Further understanding of these processes will be crucial for developing better strategies to achieve optimal osseointegration. In addition, different methods such as surface modification, systemic or local administration of certain drugs need to be optimized. Also, how to minimize the side effects should be considered in future studies.

## Data Availability

Not applicable.

## Abbreviations

ApoVs: Apoptotic vesicles
ASH1L: Absent, small, or homeotic-like 1
BIC: Bone-to-implant contact
BLCs: Bone-lining cells
BMMSCs: Bone marrow mesenchymal stem cells
CBFβ: Core binding factor subunit beta
DVL: Disheveled
EVs: Extracellular vesicles
EZH2: Enhancer of zeste homology 2
FOXP1: Forkhead box P1
H3K4: Histone 3 lysine 4
HA: Hydroxyapatite
HDAC9: Histone deacetylase 9
HOXB7: Homeobox B7
HSCs: Hematopoietic stem cells
KDM5A: Lysine (K)-specific demethylase 5A
MAF: MAF bZIP transcription factor
MAO: Microarc oxidation
miRNAs: MicroRNAs
MSCs: Mesenchymal stem cells
nHP: Nanohydroxyapatite
NF-κB: Nuclear factor κB
PDA: Polydopamine
PLGA: Poly (lactic-co-glycolic acid)
PPARγ2: Proliferator-activated receptor γ2
PTH: Parathyroid hormone
RBPjκ: Recombination signal binding protein for immunoglobulin κ J region
ROS: Reactive oxygen species
SATB2: Special AT-rich sequence-binding protein 2
SLA: Sandblasted, large grit, acid-etched
Sr: Strontium
TERT: Telomerase traverse transcriptase
TMEM64: Transmembrane protein 64
